# Properties of a Symmetrical Photoacoustic Helmholtz Cell Operating with Imbalanced Counterphase Light Stimulation

**DOI:** 10.3390/s23167150

**Published:** 2023-08-13

**Authors:** Tomasz Starecki, Michał Henryk Pietrzak, Marcin Kamil Ścisłowski

**Affiliations:** 1 Institute of Electronic Systems, Faculty of Electronics and Information Technology, Warsaw University of Technology, Nowowiejska 15/19, 00-665 Warsaw, Poland; 2 XIV LO im. Stanisława Staszica, Nowowiejska 37A, 02-010 Warsaw, Poland; 3 Faculty of Mathematics and Information Science, Warsaw University of Technology, Koszykowa 75, 00-662 Warsaw, Poland; 4 XLVIII LO im. Edwarda Dembowskiego, Barska 32, 02-315 Warsaw, Poland

**Keywords:** photoacoustic Helmholtz resonator, differential photoacoustic cell, frequency response, counterphase light stimulation

## Abstract

The output signal from a photoacoustic cell based on a symmetrical Helmholtz resonator structure can be substantially increased if a counterphase light stimulation is applied to the cell cavities. However even slight differences in the intensity of the light beams irradiating the cavities may affect the frequency response of the cell and the output signal level. This paper shows the influence of the imbalanced light irradiation on the properties of such a cell. It was found that even at relatively high irradiation mismatch, and even with the photoacoustic signal detection implemented with a single microphone, the influence of the irradiation imbalance on the frequency response of the cell around the resonance frequency is not critical. In the case of differential detection of the photoacoustic signal, the imbalance of the light irradiation does not affect the frequency response of the cell, but only the output signal level.

## 1. Introduction 

Photoacoustics is a technique with many potential applications, but among the most common are photoacoustic spectroscopy and trace gas detection [1,2,3]. The most common approach to photoacoustic detection of trace amounts of a given compound is based on the use of a light source which produces a modulated light beam of a single wavelength selected according to the substance being detected. The investigated gas sample is placed in a container (photoacoustic cell) and irradiated with the modulated light beam. As a result, a photoacoustic signal is induced, which is then converted into an electrical signal by means of a microphone or a piezoelectric transducer. In the case of photoacoustic trace gas detection applications we usually look for the highest possible sensitivity, i.e., the possibility of detecting the lowest possible concentration of the investigated compounds; one of the components which have a significant impact on this sensitivity is the design of the photoacoustic cell.

A common solution used to increase the photoacoustic signal level, and, thus, to improve the sensitivity of the setup, is the implementation of the photoacoustic cells as resonant cells with standing acoustic waves or a Helmholtz resonance [4,5,6,7,8,9,10,11,12,13,14,15,16,17]. In the case of photoacoustic cells with standing wave resonances [4,5,6,7,8], the resonance frequency is usually relatively high. This results from the fact that the length *λ* of the acoustic wave is determined by the following formula:

(1) $$\lambda =\frac{v}{f},$$

where

*v*—speed of sound propagation in the medium filling the cell,
*f*—frequency of the acoustic wave.

If the cell was used, for example, to test trace amounts of certain substances in the air, in which the speed of sound propagation at room temperature is about 340 m/s, then, for a light beam modulation frequency of 1 kHz, acoustic wavelengths of the order of 34 cm would be obtained; for a modulation frequency of 340 Hz the wavelength would be of about 1 m. As a result, resonance cells using standing wave resonance are usually quite large or have to work with the light beam modulation frequency of at least a few kHz.

**Figure 1:**
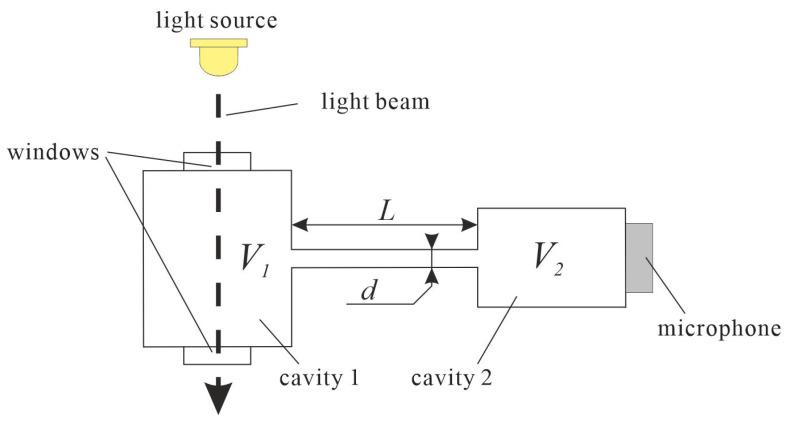
Sketch of a simple photoacoustic Helmholtz cell.

The cell can be also implemented in a form of a Helmholtz resonator. In its basic form, photoacoustic Helmholtz cell consists of two cavities connected with a duct (Figure 1). One of the cavities is equipped with a window, which allows the light to enter the interior of the cavity, interact with the investigated sample, and induce photoacoustic effect. The resulting photoacoustic signal is converted to an electronic signal with a microphone placed in the second cavity [4,5,15,16,17]. The resonant frequency *f*_0_ of such a cell is given by the approximate formula [4,5,18]

(2) $$f_{0}\approx \frac{v\varphi }{4\pi }\sqrt{\frac{\pi }{l}\frac{V_{1}+V_{2}}{V_{1}V_{2}}},$$

where

*V*_1_, *V*_2_—cavity volumes,
*l*—length of the duct connecting the cavities,
*φ*—duct diameter,
*v*—speed of sound propagation in the gas filling the cell.

As can be seen from the formula given above, the resonance frequency of the Helmholtz resonator is not a simple linear function of its dimensions as in the case of standing wave resonators, because the mechanism of resonance in such an acoustic structure is completely different. Instead of inducing a standing wave, the gas filling the Helmholtz cell moves from one of the cavities to the other and back, resulting in periodical changes of the pressure in the cavities. It should be noticed that the pressure changes in the cavities are in counterphase; i.e., while the gas pressure reaches maximum value in one of the cavities, it simultaneously reaches minimum value in the other cavity. 

Even a brief analysis of the construction of the Helmholtz resonator shows that it has several advantages from the point of view of photoacoustic applications. One of its valuable features is that it allows the use of high-sensitivity microphones with a diameter of 1/2 inch or even 1 inch, while maintaining a relatively small cell volume (in the order of single cm^3^ or even less) [19,20,21]. And a small volume of the cell leads to a relatively high output signal level due to the 1/*V* relationship of the photoacoustic signal amplitude [18]:

(3) $$A\propto \frac{P_{0}\beta \eta Q}{Vf},$$

where

*β*—absorption coefficient,
*P*_0_—light power used to excite the photoacoustic effect,
*f*—modulation frequency of the light beam,
*V*—volume of the photoacoustic cell,
*η*—efficiency of the detector (e.g., microphone),
*Q*—quality factor of the cell (if the modulation frequency of the light beam corresponds to the acoustic resonance frequency of the cell).

As can be seen from the above formula, the photoacoustic signal can be increased by the use of resonance properties of the cell (influence of the Q-factor), by lowering the frequency of the light beam modulation and/or by lowering the volume of the cell.

Practically obtained Q-factor values of the photoacoustic Helmholtz cells are not very high and typically range from a few to a dozen [22,23,24], but there are reports of photoacoustic Helmholtz cells with the Q-factor values of over twenty [25,26]. Although such values may be considered low, even a low Q-factor increases the sensitivity of the photoacoustic device. It should be also mentioned that the Q-factors of standing wave photoacoustic resonators are usually only slightly higher in comparison to Helmholtz cells.

The relationship determining the resonant frequency of a Helmholtz resonator (given by Equation (2)) shows that even with small dimensions (resulting, in particular, in small volume of the cell) it is possible to obtain relatively low resonant frequencies, e.g., of the order of a few hundred hertz, that allows for a further increase in the level of the output signal from the cell, due to the already mentioned 1/*f* relationship of the photoacoustic signal amplitude (see Equation (3)). 

## 2. Differential Photoacoustic Helmholtz Cells

**Figure 2:**
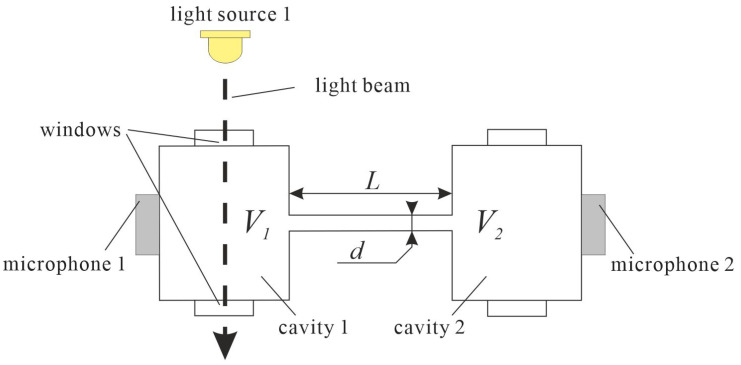
Symmetrical Helmholtz cell with differential signal detection.

The two-cavity structure of the Helmholtz cell allows not only for easy shaping of the frequency response of the cell but also enables simple implementation of differential circuits. In such a case the microphones should be placed in both cavities of the cell, and signals from the microphones should be subtracted (Figure 2) [10,11,12,13,14,27,28,29,30,31]. Such a solution allows for cancelling or at least substantially decreasing the influence of the external acoustic noise, because external acoustic noise produces similar signal compounds in both microphones, which is then cancelled or at least strongly reduced in the differential electronic setup (which subtracts these signals). On the other hand, taking into consideration that at the resonance frequency the photoacoustic signal compounds detected by the microphones are in counterphase, subtracting the signals from the microphones results in approximately doubling the level of the signal at the output of the differential circuit.

It is obvious that the best results in the use of a differential setup can be obtained if the cell used in differential configuration is developed as a symmetrical structure. This is due to the fact that in such a case the external acoustic noise propagates in an identical or at least a very similar manner to both microphones, reaching them with the same phase and amplitude, while nonsymmetrical cell design may result in different propagation of the external noise, that would lead to differences in phase and amplitude of the noise signal compounds reaching the microphones, and finally result in much worse external acoustic noise attenuation. 

**Figure 3:**
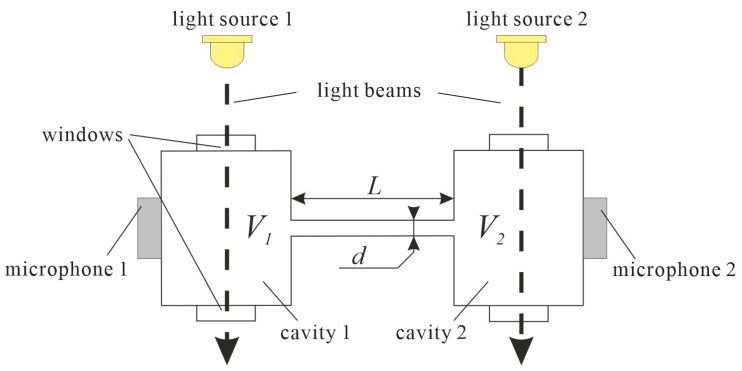
Symmetrical Helmholtz cell with differential signal detection and counterphase light stimulation.

Quite often the primary goal of a photoacoustic setup design is to obtain its maximum sensitivity. Thus, design efforts focus on obtaining high levels of the induced photoacoustic signal; according to Equation (3), this can be achieved by lowering the modulation frequency or the cell volume and by increasing the quality factor or the light power. In the case of a differential Helmholtz resonator (e.g., as presented in Figure 2) it is possible to substantially increase the output signal if the light irradiates both cavities and the light beams are modulated in counterphase (Figure 3). So far, such a solution has not been used too often [28,29,31] but is definitely worth further investigations. If both light sources have identical optical output power, such an approach should double the photoacoustic signal level compared to a single light source solution. This occurs since at the Helmholtz resonance amplitude of the signal in both cavities is identical; just the phase is opposite. Thus, at the resonance frequency the photoacoustic signal compounds coming from the light sources driving the cavities in counterphase have the same phase, so that their superposition is as a matter-of-fact addition of two signals of identical amplitude and phase.

Ideally, in a solution as given in Figure 3 the light sources should emit the same amount of power, so that samples in both cavities are irradiated equally. However, if two separate light sources are used for this purpose, this condition is usually not fulfilled, as the light sources usually show some differences in light emission efficiency. Obviously, even a slight difference in the irradiating light power results in an imbalance of the signals induced in the two cavities, thus changing the properties of the setup. This work aimed to investigate how much such an imbalance affects the frequency response of a cell, and how critical for expected properties and proper operation of the photoacoustic setup is to keep the light beam intensity equal.

## 3. Theoretical Analysis

**Figure 4:**
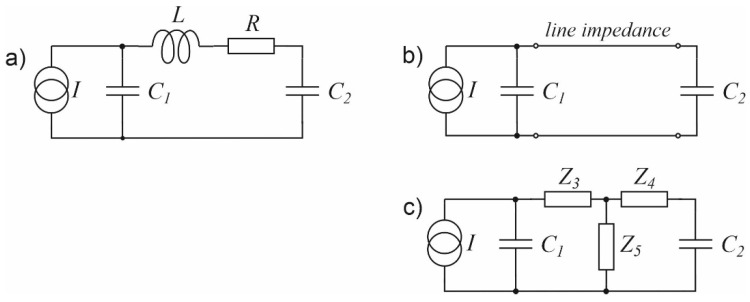
Electric models of a simple photoacoustic Helmholtz cell: (**a**) With a lossy inductance representing properties of the duct connecting the cavities; (**b**) With the duct modelled with a transmission line; (**c**) With the transmission line substituted with an equivalent T-section with lumped impedances.

The most commonly used technique for modelling of photoacoustic Helmholtz cells is the acoustoelectrical analogy method, in which each acoustic element of the cell is replaced with its corresponding electrical component [32,33,34]. In the simplest model of the cell shown in Figure 1, there are only a few lumped elements (Figure 4a)—capacitors modelling the cavities, a lossy inductance which is the equivalent of a channel connecting the cavities, and a current source whose task is to reflect excitations resulting from the photoacoustic effect occurring in the cavity containing irradiated sample. Although the presented model is very simple, the problems regarding this model appear already at the stage of defining the values of components used in such a model. It turns out that the values of inductance *L* and loss resistance *R* can be defined in at least several ways. For example, Morse [32] defined the values of these elements as

(4) $$L=\frac{\rho l}{\pi a^{2}},\;\;\;\;\;R=\frac{{\rho \omega }^{2}}{2\pi v},$$

Blitz [33,34] assumed

(5) $$L=\frac{\rho l}{\pi a^{2}},\;\;\;\;\;R=\frac{8\eta l}{\pi a^{4}},$$

Nolle [35] used

(6) $$L=\frac{4}{3}\frac{\rho l}{\pi a^{2}},\;\;\;\;\;R=\frac{8\eta l}{\pi a^{4}},$$

Kästle and Sigrist [23] applied

(7) $$L=\frac{\rho l}{\pi a^{2}},\;\;\;\;\;R=\frac{l\sqrt{2\rho \omega }}{\pi a^{3}}\left(\sqrt{\eta }+\sqrt{\frac{k_{g}}{c_{p}}}\left(\frac{c_{p}}{c_{v}}-1\right)\right),$$

while Mattiello et al. [26] in some cases assumed

(8) $$L=\frac{4\rho l}{\pi a^{2}},\;\;\;\;\;R=l\sqrt{\frac{2\rho \omega \eta }{\pi a^{3}}}.$$

The variables in the above definitions are as follows:

*ω*—angular frequency of the light beam modulation and induced photoacoustic signal,
*a*—radius of the channel connecting the cavities,
*l*—channel length,
*c_p_*—specific heat of gas at constant pressure,
*c_v_*—specific heat of gas at constant volume,
*ρ*—gas density,
*k_g_*—thermal conductivity of the gas,
*η*—gas viscosity,
*v*—speed of sound propagation in the gas filling the cell.

In fact, none of the above definitions gives satisfactory results [36,37]. The measured values of the resonance frequency of such cells sometimes differ by as much as several dozen percent from the values obtained using simulations, and the ratios of the modelled to the measured Q-factors often reach the order of several dozen to several hundred [36,37,38]. Therefore, the sense of using such models becomes highly questionable.

A much better agreement with the measurements of the actual photoacoustic characteristics of Helmholtz cells can be obtained by replacing the model with lumped components with a transmission line model (Figure 4b) [39,40], whose properties much better reflect the influence of viscous and thermal interactions of the gas during its flow through the channel connecting the cavities. Although it is commonly stated that modelling with distributed elements should be used only when the physical size of elements is at least comparable to the signal wavelengths, in the case of photoacoustic Helmholtz cells transmission line models give much better approximation than lumped-element models even in the case of very short ducts connecting the cavities [36,38].

In the transmission line model, the characteristic impedance of the line and the propagation constant are defined as follows:

(9) $$Z_{f}=\sqrt{\frac{R+j\omega L}{G+j\omega C}},\;\;\;\;\;\Gamma =\sqrt{\left(R+j\omega L\right)\left(G+j\omega C\right)},$$

where [41]

(10) $$R+j\omega L=j\frac{\omega \rho }{{\pi a}^{2}\left(1-F_{v}e^{{j\Phi }_{v}}\right)},$$

(11) $$G+j\omega C=j\frac{\omega \pi a^{2}}{\rho c^{2}}\left(1+\left(\kappa -1\right)F_{t}e^{{j\Phi }_{t}}\right).$$

The symbol *κ* in the above relations denotes the adiabatic coefficient of the gas filling the cell (*κ = c_p_/c_v_*), while the function *F* is given as

(12) $$F_{v,t}e^{{j\Phi }_{v,t}}=\frac{2J_{1}\left(\sqrt{-j}r_{v,t}\right)}{\sqrt{-j}r_{v,t}J_{0}\left(\sqrt{-j}r_{v,t}\right)},$$

where *J*_0_ and *J*_1_ are complex zero- and first-order Bessel functions, and the subscripts *v* and *t* in equations. Equations (10) and (11) determine which of the variables *r_v_* or *r_t_*

(13) $$r_{v}=a\sqrt{\frac{\omega \rho }{\eta }},\;\;\;\;\;\;r_{t}=a\sqrt{\frac{\omega \rho c_{p}}{k_{g}}}$$

should be used in place of the symbol *r_v_, _t_* in the expression Equation (12).

The transmission line described above can obviously be represented by a T-section (Figure 4c), where the impedances *Z*_3_, *Z*_4,_ and *Z*_5_ are defined as follows [42]:

(14) $$Z_{3}=Z_{4}=Z_{f}\tan h\frac{\Gamma l}{2},{\;\;\;\;\;Z}_{5}=\frac{Z_{f}}{\sin h\left(\Gamma l\right)},$$

where *l* is the length of the channel, and values of the capacitances modelling the cavities are calculated from the formula [34,35,36].

(15) $$C_{i}=\frac{V_{i}}{{\rho v}^{2}},$$

where *V_i_* is the volume of the *i*-th cavity (*i* = 1, 2).

**Figure 5:**
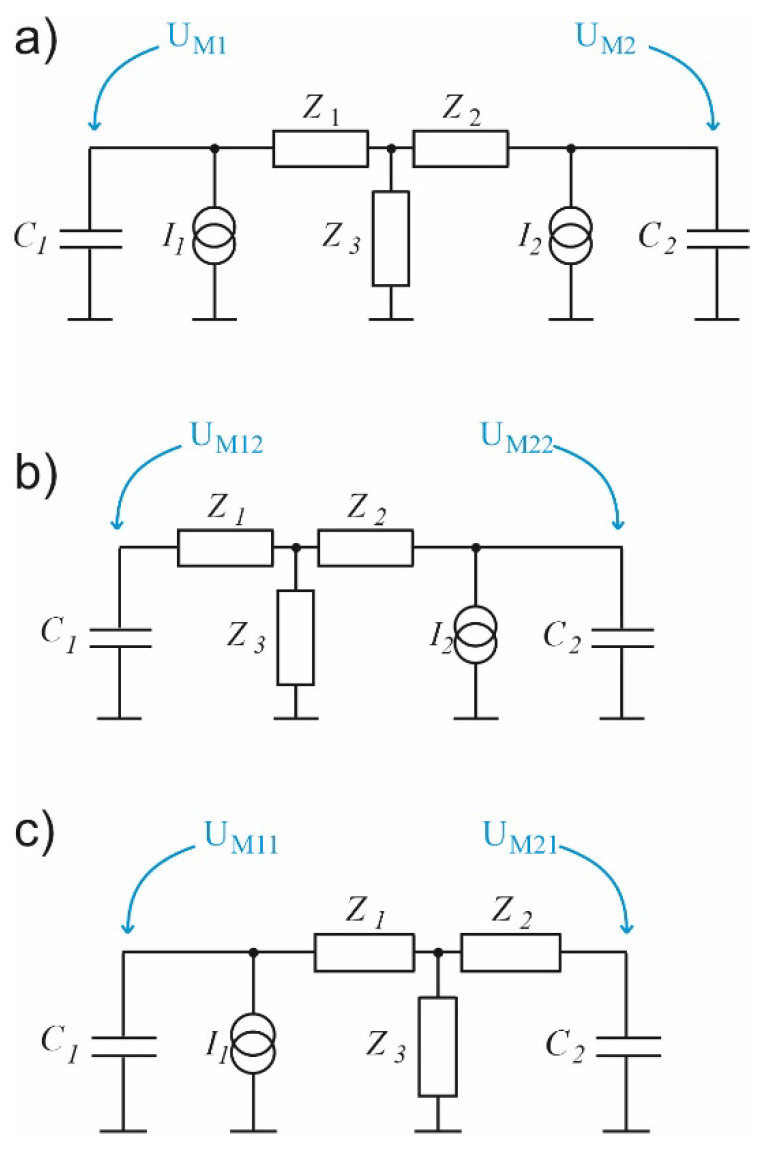
Transmission line model of the symmetrical photoacoustic Helmholtz cell with differential signal detection: (**a**) With counterphase light stimulation; (**b**) With only light source 1 active; (**c**) With only light source 2 active.

Taking the above into consideration, a cell with the structure as given in Figure 3 can be modelled using the circuits presented in Figure 5, where I1 and I2 current sources are used to model the intensity of the light beams which irradiate the cell cavities, while U_M1_ and U_M2_ correspond to pressure changes in these cavities and, thus, to voltage signals at the outputs of the microphones placed in the cell [39]. The signal resulting from simultaneous operation of both light sources can be calculated as a superposition of the signals produced by two individual light sources (as shown at Figure 5b,c), so that U_M1_ = U_M11_ + U_M12_ and U_M2_ = U_M21_ + U_M22_. Such models have already been tested and found to produce very good results in modelling photoacoustic Helmholtz cells and multicavity structures as well [28,40].

**Figure 6:**
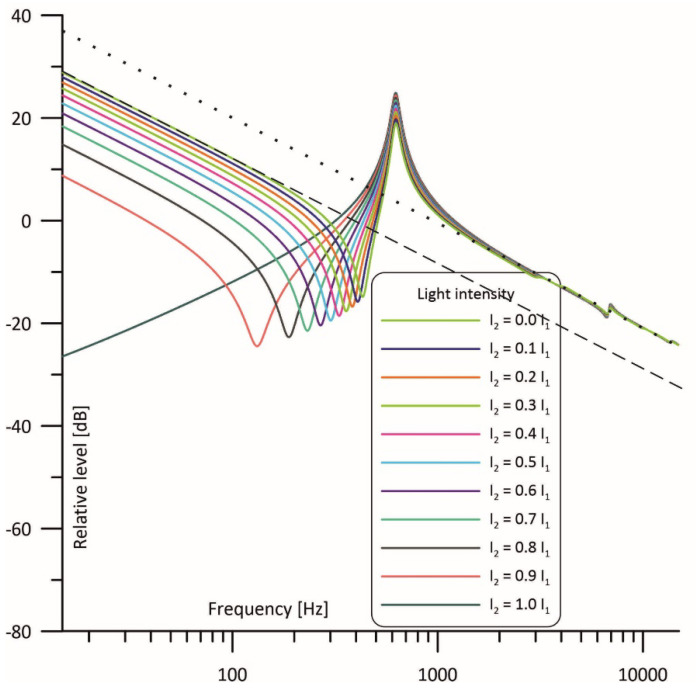
Simulated frequency response of the cell with the intensity of one light beam fixed and of the other light beam varied from 0 to 100% observed in the cavity with the fixed power of irradiation.

**Figure 7:**
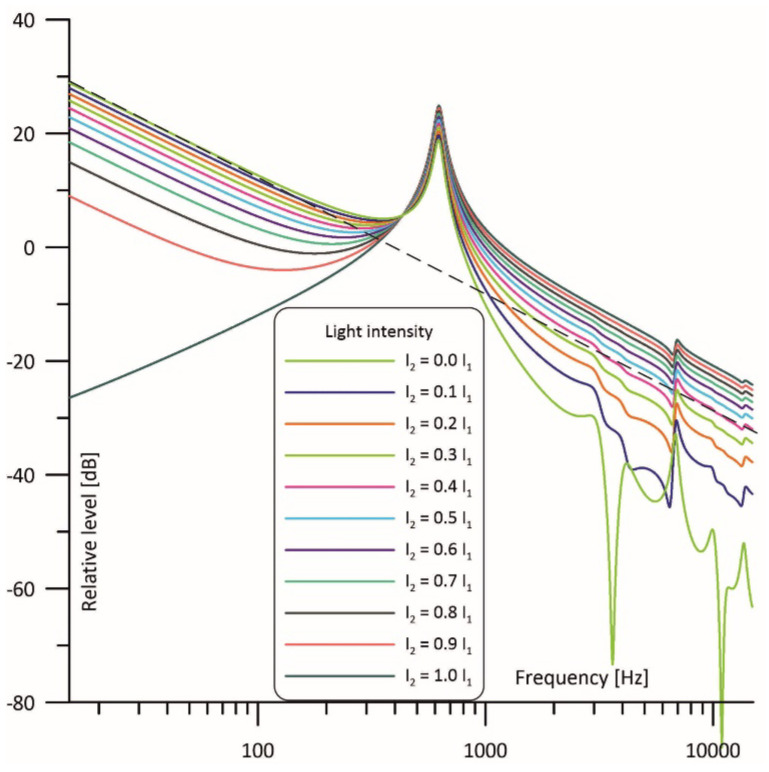
Simulated frequency response of the cell with the intensity of one light beam fixed and of the other light beam varied from 0 to 100% observed in the cavity with the variable power of irradiation.

**Figure 8:**
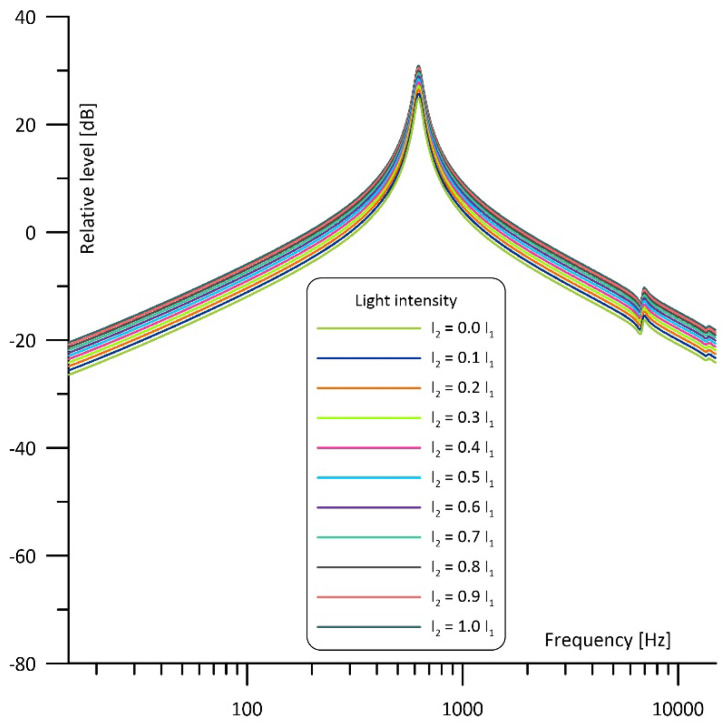
Simulated frequency response of the cell with the intensity of one light beam fixed and of the other light beam varied from 0 to 100% operating with differential signal detection.

Results obtained from the simulations are presented in Figure 6, Figure 7 and Figure 8 and show how much change in the light intensity of one of the light beams affects the frequency response of the photoacoustic cell. Figure 6 shows frequency responses of the cell observed from the cavity in which the power of the light is assumed to be held at a stable level considered as 100%, while the power of the diode irradiating the second cavity is being changed from 0% to 100%, respectively. When looking at the curve for I_2_ = 0 I_1_ it should be noticed that at low frequencies the resonance properties of the Helmholtz cell can be neglected (influence of the duct on the gas flow is so low that pressure is virtually at the same level in both cavities). As a result, the curve shows 1/f behavior like for a nonresonant cell in which volume is equivalent to the sum of volumes of both cavities’ behavior (see the dashed line). At medium frequencies a Helmholtz resonance is observed. At higher frequencies (above the resonance) acoustic impedance of the interconnecting duct increases significantly, and the gas flow between the cavities is dramatically reduced. As a result, the sample cavity behaves nearly like a nonresonant cell of the volume equivalent to the single cavity volume, showing again 1/f behavior but at a slightly level higher than below the resonance (see the dot line). For the same reason (high acoustic impedance of the duct resulting in a very limited gas flow), an increase in I_2_ has virtually no influence on the frequency response curves above the resonance. 

Interesting changes in the frequency responses can be observed below the resonance frequency. Gradual increase of I_2_ results in shifting antiresonance to the left (toward lower frequencies) and decreases the level of the photoacoustic signal at the frequencies below the antiresonance. At first this may be considered undesirable, assuming that in such a case working at lower modulation frequencies would result in lowering the output signal. But such conclusions would be incorrect, as we should take into consideration that we use a differential Helmholtz cell when we intend to apply it in resonance operation, and at the resonance increase of I_2_ results in the rise of the output photoacoustic signal. Finally, for I_2_ = I_1_ the obtained curve exhibits narrowband filtering properties around the resonance, which is strongly advantageous as it allows for better filtering of any unwanted signals, in particular, any kind of acoustic noise.

Importantly, even a 10% difference in the light intensity has considerable influence on the frequency response of the cell. However, it is not critical if we care only about the shape of the frequency response which is close to the resonance. 

A similar situation is observed in Figure 7, which shows frequency responses of the cell observed in the other cavity. Again, when looking at the curve for I_2_ = 0 I_1_ at low frequencies, resonance properties of the Helmholtz cell can be neglected, and we can observe 1/f behavior (see the dashed line) like in the case of a nonresonant cell. At medium frequencies a Helmholtz resonance is observed. At higher frequencies (above the resonance) acoustic impedance of the interconnecting duct increases significantly, and the gas flow between the cavities is dramatically reduced. But, contrary to what can be seen in Figure 6, this means that the photoacoustic signal induced in the irradiated cavity is strongly attenuated before getting to the microphone placed in the other cavity. Thus, the frequency response above the resonance region falls below the dashed line. An increase in I_2_ decreases the level of the photoacoustic signal at the frequencies below the resonance while raising the photoacoustic signal at the frequencies above the resonance; finally, for I_2_ = I_1_ we obtain exactly the same curve as in Figure 6. Similarly to what we can observe in Figure 6, even a small difference in the light intensity has a noticeable influence on the frequency response, but the shape of the frequency response close to the resonance remains relatively stable. 

The most interesting simulation results were obtained for the differential operation of the cell, when signals from the microphones were subtracted (Figure 8). In such a case the imbalance of the light beams’ intensity has an influence on the signal level only, while the shape of the frequency response curve is kept unaffected. In this configuration narrowband filtering properties result from differential signal detection applied with a symmetrical acoustic structure of the cell and (if the light sources operate in counterphase) the induced photoacoustic signal is proportional to the sum of the light beams’ power. Thus, if I_2_ = 1.0 I_1_ the photoacoustic signal will be doubled in comparison to the case when I_2_ = 0. 

It is worth noticing that if both light sources are well matched in terms of the output optical power then the frequency response shows the same narrowband filtering properties and has exactly the same shape as in the case of the differential signal detection even if only one microphone is used (compare I_2_ = 1.0 I_1_ curves in the Figure 6, Figure 7 and Figure 8).

## 4. Measurement Results

**Figure 9:**
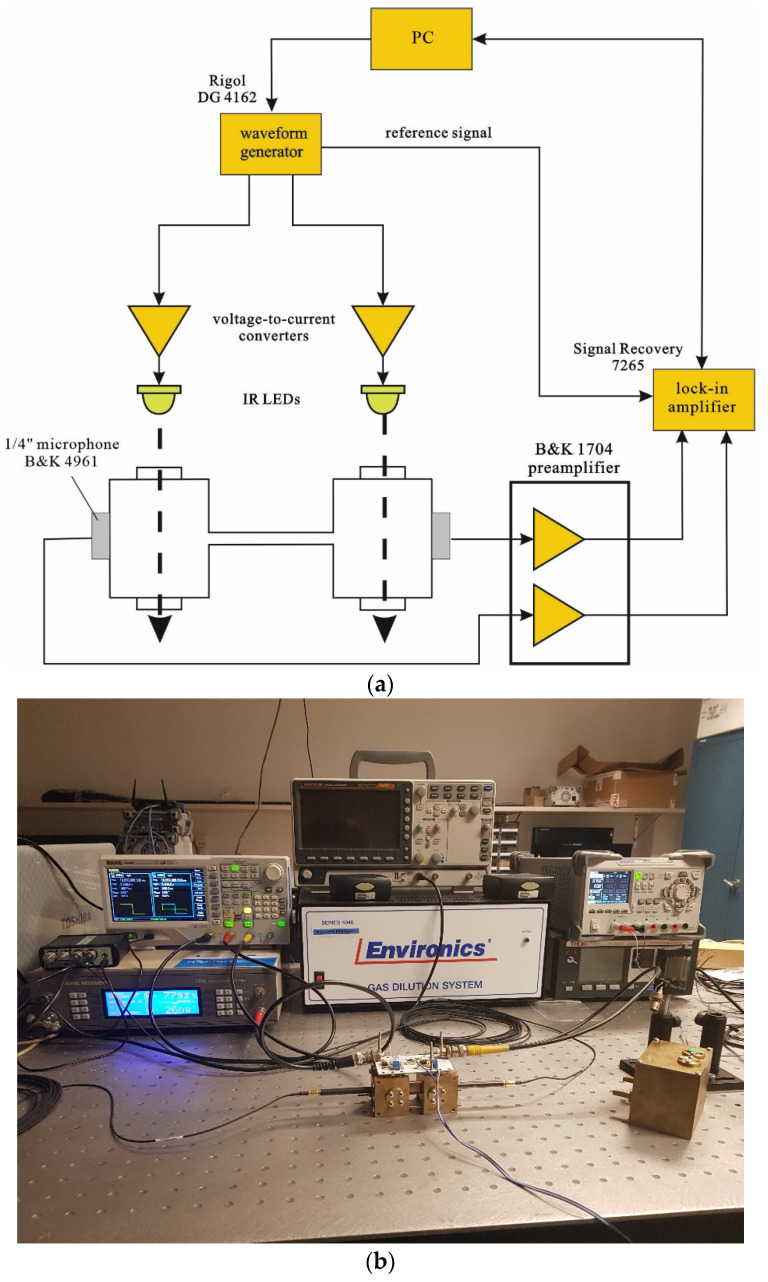
Experimental setup used for cell properties measurements: (**a**) Block diagram; (**b**) Photo of the system.

The measurements were performed in a system presented in Figure 9. In order to precisely control two IR LEDs serving as the light sources inducing a photoacoustic effect in the cell cavities, a two-channel function generator (DG 4162 Rigol [43]) driving two simple homemade voltage–current converters was used. Each channel was used to control one of the two IR LED diodes. The diodes were positioned right above the cell windows, with a very small spacing (1–2 mm) preventing the mechanical stress of the LED package (resulting from the current flow) from being transmitted to the body of the cell. The body of the cell was made of brass. In order to obtain a high level of the photoacoustic signal, carbon black was used as the light absorbing substance. The photoacoustic signal was sensed by means of two high-sensitivity (60 mV/Pa) 1/4-inch microphones (B&K 4961 [44]). Signals from the microphones were amplified in a two-channel signal conditioner (B&K 1704 [45]) and then supplied to a digital lock-in amplifier (Signal Recovery 7265 [46]). The whole system was controlled from a PC, which was also used for data recording. Frequency responses of the cell were measured in the same configurations as used during simulations.

**Figure 10:**
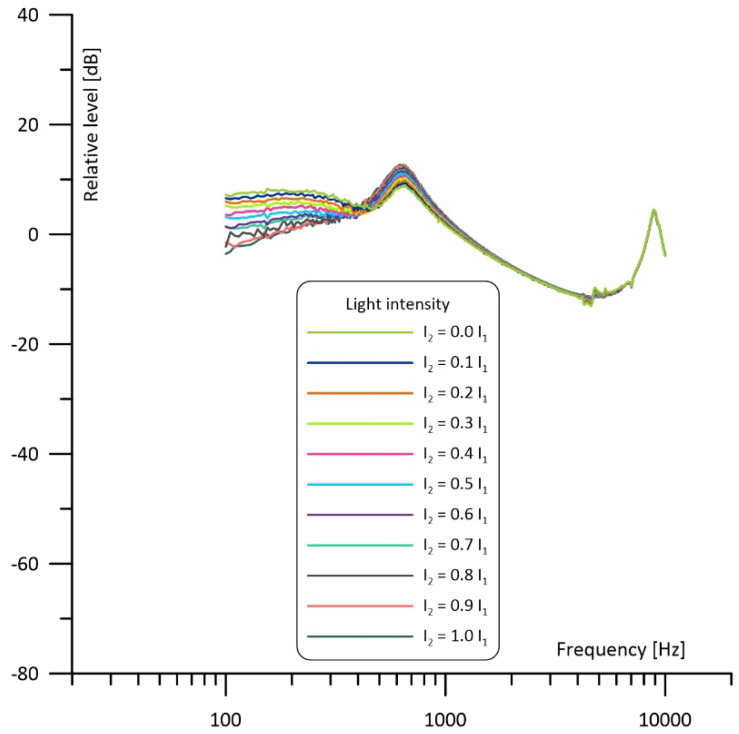
Measured frequency response of the cell with the intensity of one light beam fixed and of the other light beam varied from 0 to 100% observed in the cavity with the fixed power of irradiation.

**Figure 11:**
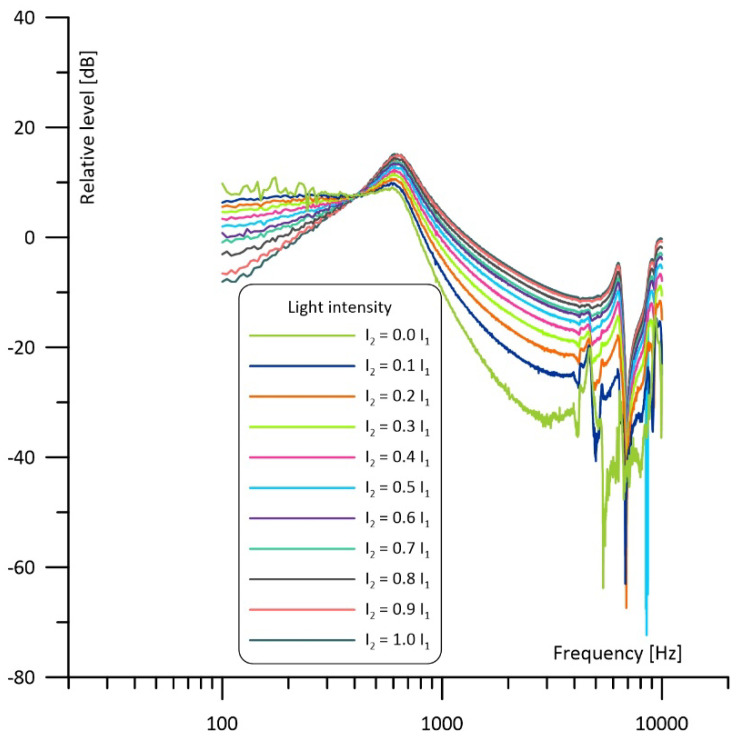
Measured frequency response of the cell with the intensity of one light beam fixed and of the other light beam varied from 0 to 100% observed in the cavity with the variable power of irradiation.

**Figure 12:**
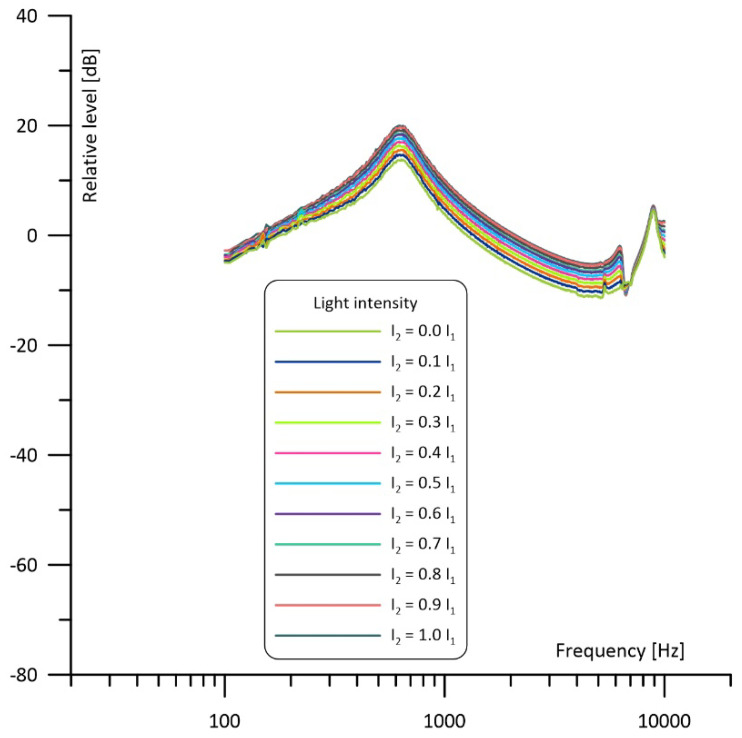
Measured frequency response of the cell with the intensity of one light beam fixed and of the other light beam varied from 0 to 100% operating with differential signal detection.

**Figure 13:**
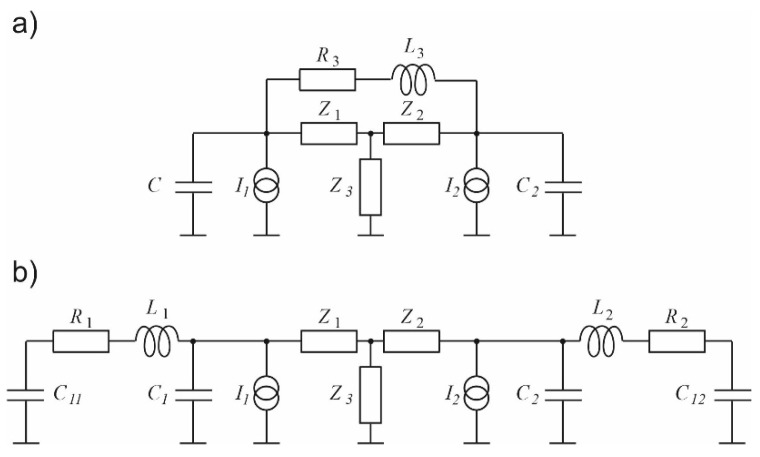
Extended models that would allow for better explanation of the measurement results: (**a**) Model the components simulating cross-talks between the cavities; (**b**) Model with the components describing leakage from the cavities to the exterior of the cell.

The results of the measurements are presented in Figure 10, Figure 11 and Figure 12. Comparison with the simulations presented in Figure 6, Figure 7 and Figure 8 shows three main differences: the measurements show no 1/f behavior at the frequencies below about 250 Hz (compare Figure 6 and Figure 10), the shape of the frequency responses above 4 kHz is slightly different (compare Figure 7 and Figure 11), and the measured narrowband filtering properties around the main Helmholtz resonance are a bit worse than theoretical (compare Figure 8 and Figure 12). Lack of the 1/f behavior can result from at least two sources: partial sound propagation (cross-talks) through the metal body of the cell or imperfect sealing of the cell (due to the lack of O-rings between the microphones and the cell). Models that would include such factors are presented in Figure 13. The crosstalk path between the cavities can be modelled using a lossy inductance (components L_3_, R_3_ in Figure 13a). Acoustic leakage between the cavities and exterior of the cell can be also simulated using lossy inductances (components L_1_, R_1_ and L_2_, R_2_ in Figure 13b) while capacitances C_11_ and C_12_ are used to model the exterior of the cell. It should be mentioned that in all these cases the resistances (R_3_ in Figure 13a and R_1_, R_2_ in Figure 13b) play the dominant role. Under appropriate selection of the mentioned components, both presented models would allow for obtaining simulation results similar to the measured frequency responses. Moreover, presence of the losses introduced by the resistances would also explain the lower Q-factor of the cell (worse narrowband filtering properties). Differences at higher frequencies (above 4 kHz) in comparison to simulations may result from some standing wave resonances which are not modelled with the technique used during the simulations. Despite all the mentioned differences between the simulations and the measurement results, it is clearly visible that the experimental results are in good agreement with the simulations in a relatively wide range of the frequencies around the resonance. The most important is that, except from lower value of the Q-factor, the measured frequency responses obtained for differential operation of the cell (Figure 12) were in nearly perfect agreement with the simulation results (Figure 8).

## 5. Conclusions

According to the obtained simulation and experimental results we can state that the application of a symmetrical photoacoustic differential Helmholtz cell working with two light sources which operates with counterphase light stimulation has strong advantages of narrowband filtering properties and doubling the photoacoustic signal level, resulting in higher detection sensitivity of the setup. Certainly, even relatively small differences in light intensity of the diodes affect the frequency response of a cell. This is important information as, due to production process variation, even LED or laser diodes of the same model run with the same amount of current differ in their optical output powers. Similarly, even slightly different positioning of a diode can result in different level of irradiation of the cavity interior. All these factors contribute to the imbalance of the irradiation of the cell cavities. Obviously, optimal situation is when intensities of the light beams entering both cell cavities are identical. However, it turns out that the influence of the irradiation imbalance on the frequency response of the cell around the Helmholtz resonance frequency is not critical even at noticeable irradiation mismatch, e.g., at the level of 10%, and even if the cell is used in a single microphone configuration. Thus, precise adjustment of the light sources in order to obtain very well-matched values of their optical emission is not required. Moreover, in the case of differential detection of the photoacoustic signal, the imbalance of the light irradiation does not affect the frequency response of the cell but only the output signal level. 

## Data Availability

On request from the authors.
